# Tildrakizumab: A new therapeutic option for erythrodermic psoriasis?

**DOI:** 10.1111/dth.15030

**Published:** 2021-06-22

**Authors:** Matteo Megna, Luca Potestio, Gabriella Fabbrocini, Eleonora Cinelli

**Affiliations:** ^1^ Section of Dermatology, Department of Clinical Medicine and Surgery University of Naples Federico II Napoli Italy

## CONFLICT OF INTEREST

The authors declare no potential conflict of interest.

Dear Editor,

Erythrodermic psoriasis (EP) is a life‐threatening condition characterized by a severe erythema affecting at least 80%–90% of the body surface area.^1^ Systemic symptoms such as fever, tachycardia, lymphadenopathy, arthralgia and fatigue can be associated with EP.^1^

**FIGURE 1:**
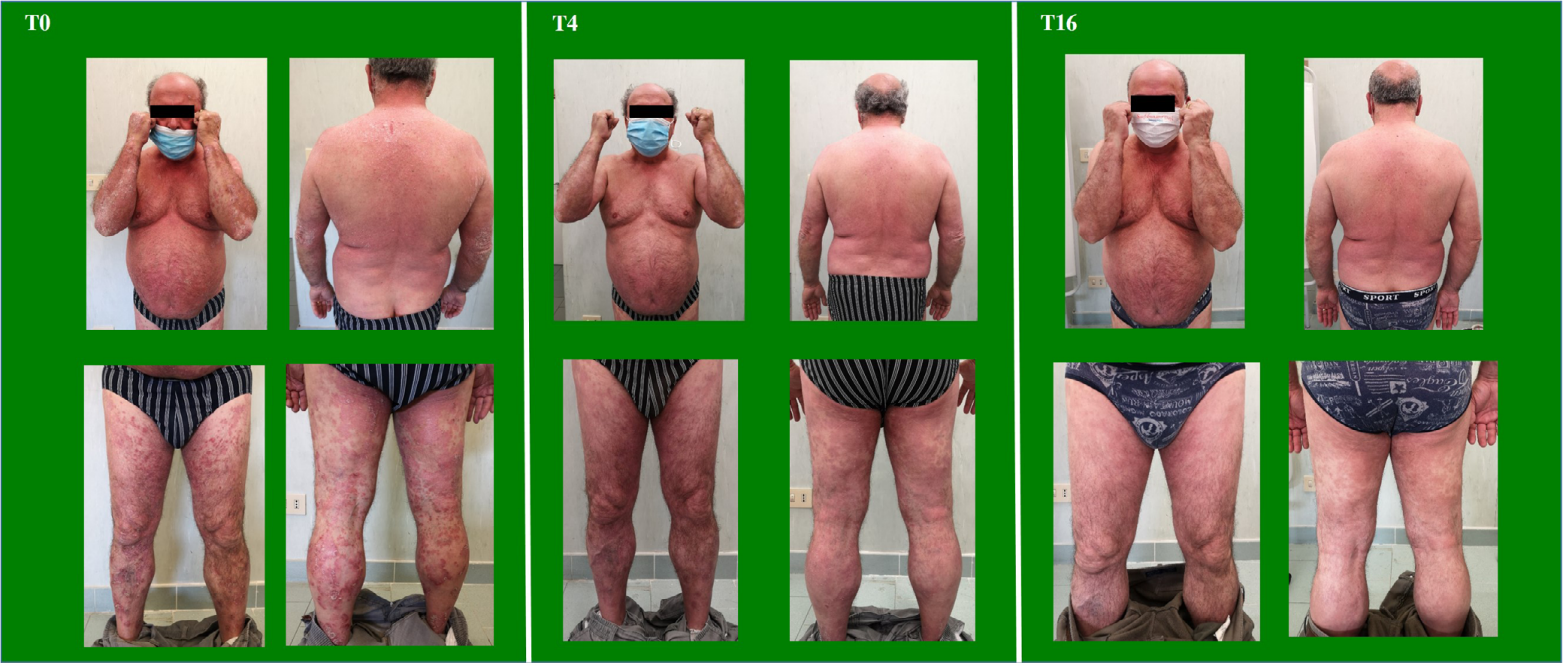
Patient at baseline (T0), 4‐week (T4), and 16‐week (T16) follow‐ups

Here we describe a case of a 61‐year‐old man referring to our Department in September 2020. The patient reported a sudden worsening of his long‐duration psoriasis (35 years): on clinical examination skin lesions were extended to 90% of body surface area (BSA), for a total Psoriasis Area Severity Index (PASI) of 38 (Figure 1). He also complaint of fatigue and chills. In addition, the patient suffered from hypertension treated with ace inhibitors and obesity. Previous psoriasis treatments included only topicals and heliotherapy. Because of the severity of the disease, the disabling impact on the patient's quality of life and the patient's refusal to conventional therapy for safety concerns, he was screened for biological therapy.

In order to increase patient's compliance to treatment and to give an effective and safe therapy, Tildrakizumab was chosen, due to the lowest number of administrations required among biologics (1 s.c. injection every 12 weeks after the induction phase), as requested from the patient.

Tildrakizumab at labeled dosage was started (100 mg at weeks 0, 4, and every 12 weeks thereafter). At 4‐week follow‐up, PASI decreased to 7.8 and BSA to 25%; PASI75 response was rapidly reached (Figure 1). At week 16, a complete resolution of skin lesions (PASI100) was achieved (Figure 1), along with a substantial improvement of the fatigue symptom.

Before the introduction of biologic therapy, first‐line options for the treatment of EP were based on traditional systemic therapy: methotrexate, acitretin, and cyclosporine.^2^ Later on, the introduction of biologics has provided for faster and safer results for EP: indeed, several case reports support the use of biologic therapy as first‐line treatment for EP.^3^ Although the severity and the rarity of this condition do not allow the inclusion of EP patients in clinical studies, the current literature is supplying evidences from real world experiences (RWEs). Currently, as pointed out by a systematic review, positive responses have been described in cases treated by different classes of biologics, from anti‐TNF‐α to anti‐IL23.^3^, ^4^ Up until now, no case describing EP successfully treated by the latest anti‐IL23 approved in Europe,^5^ Tildrakizumab, has been described. Here we firstly report a case of a 61‐year old man with EP successfully and safely treated by Tildrakizumab. A significant improvement was experienced since the first follow up at week 4, thus giving the patient the possibility to benefit from a better quality of life in a short time of treatment. Although our study reports only a single case of EP treated by Tildrakizumab, further experiences could widen the therapeutical horizons of biologics, even the latest ones, to evaluate their efficacy and safety for EP. In conclusion, our case is part of the literature reports from RWEs that could have a significant role to establish the best therapeutic algorithm and tailored options for EP patients.

## Data Availability

Data sharing not applicable to this article as no datasets were generated or analyzed during the current study.
